# Evolution of patients’ socio-behavioral characteristics in the context of DAA: Results from the French ANRS CO13 HEPAVIH cohort of HIV-HCV co-infected patients

**DOI:** 10.1371/journal.pone.0199874

**Published:** 2018-07-05

**Authors:** Issifou Yaya, Perrine Roux, Fabienne Marcellin, Linda Wittkop, Laure Esterle, Bruno Spire, Stéphanie Dominguez, Boni Armand Elegbe, Lionel Piroth, Philippe Sogni, Dominique Salmon-Ceron, Maria Patrizia Carrieri, the ANRS CO13 HEPAVIH Study Group

**Affiliations:** 1 Aix Marseille Univ, INSERM, IRD, SESSTIM, Sciences Economiques & Sociales de la Santé & Traitement de l’Information Médicale, Marseille, France; 2 ORS PACA, Observatoire régional de la santé Provence-Alpes-Côte d’Azur, Marseille, France; 3 Univ. Bordeaux, ISPED, INSERM, Bordeaux Population Health Research Center, team MORPH3EUS, UMR 1219, CIC-EC 1401, Bordeaux, France; 4 CHU de Bordeaux, Pole de sante publique, Service d’information medicale, Bordeaux, France; 5 INSERM U955, AP-HP, Groupe Henri-Mondor Albert-Chenevier, Immunologie Clinique et Maladies Infectieuses, Créteil, France; 6 Département d'Infectiologie, Centre Hospitalier Universitaire and INSERM CIC 1432, Université de Bourgogne, Dijon, France; 7 Université Paris Descartes, INSERM U-1223, Institut Pasteur, Service d'Hépatologie, hôpital Cochin, Assistance Publique–Hôpitaux de Paris, Paris, France; 8 Université Paris Descartes, Service Maladies Infectieuses et Tropicales, AP-HP, Hôpital Cochin, Paris, France; Centers for Disease Control and Prevention, UNITED STATES

## Abstract

**Background:**

Direct-acting antivirals (DAA) have dramatically increased HCV cure rates with minimal toxicity in HIV-HCV co-infected patients. This study aimed to compare the socio-behavioral characteristics of patients initiating pegylated-interferon (PEG-IFN)-based HCV treatment with those of patients initiating DAA-based treatment.

**Methods:**

ANRS CO13 HEPAVIH is a national multicenter prospective cohort started in 2005, which enrolled 1,859 HIV-HCV co-infected patients followed up in French hospital outpatient units. Both clinical/biological and socio-behavioral data were collected during follow-up. We selected patients with socio-behavioral data available before HCV treatment initiation.

**Results:**

A total of 580 patients were included in this analysis. Of these, 347 initiated PEG-IFN-based treatment, and 233 DAA-based treatment. There were significant differences regarding patient mean age (45 years±6 for the PEG-IFN group vs. 52 years±8 for the DAA group, p<0.001), unstable housing (21.4% vs. 11.2%, p = 0.0016), drug use (44.7% vs. 29.6%, p = 0.0003), regular or daily use of cannabis (24.3% vs. 15.6%, p = 0.0002), a history of drug injection (68.9% vs 39.0%, p<0.0001) and significant liver fibrosis (62.4% vs 72.3%, p = 0.0293). In multivariable analysis, patients initiating DAA-based treatment were older than their PEG-IFN-based treatment counterparts (aOR = 1.17; 95%CI [1.13; 1.22]). Patients receiving DAA treatment were less likely to report unstable housing (0.46 [0.24; 0.88]), cannabis use (regular or daily use:0.50 [0.28; 0.91]; non-regular use: 0.41 [0.22; 0.77]), and a history of drug injection (0.19 [0.12; 0.31]).

**Conclusion:**

It is possible that a majority of patients who had socio-economic problems and/or a history of drug injection and/or a non-advanced disease stage were already treated for HCV in the PEG-IFN era. Today, patients with unstable housing conditions are prescribed DAA less frequently than other populations. As HCV treatment is prevention, improving access to DAA remains a major clinical and public health strategy, in particular for individuals with high-risk behaviors.

## Introduction

Chronic hepatitis C virus (HCV) infection is an endemic disease that affects approximately 71 million people worldwide [1], with significant regional variability. In recent decades, great progress has been made in treating chronic HCV infection. First-generation treatments were mainly composed of pegylated interferon (PEG-IFN) and ribavirin and were administered for at least 48 weeks. The response rate was 60–70% for genotypes 2 and 3 but lower for genotypes 1 and 4 [2]. Moreover, PEG-IFN-based treatments had significant side effects including flu-like symptoms, asthenia, neuropsychiatric symptoms, hematological toxicity, rashes, and more lifetime complications [3,4].

The recent emergence of a new generation of treatments—direct-acting antivirals (DAA)–constitutes a revolution in HCV care. DAA are more effective and better tolerated than PEG-IFN and ribavirin, and in 8 to 24 weeks a sustained virological response (SVR) is obtained in over 90% of treated patients, including previously difficult-to-treat patients and HIV-HCV co-infected patients [5–8].

However, the costs of DAA are very high, and access varies across global regions. Even in countries with a universal health system, restricted access is not uncommon [9,10]. Germany and the United Kingdom are two countries that immediately implemented universal access to DAA treatment [11], and all patients infected with HCV are treated unconditionally. In both the PEG-IFN and DAA eras, in some countries, the most common barriers to initiating HCV treatment in HIV-HCV co-infected patients include psychoactive substance use, and social instability such as homelessness or imprisonment [12].

In May 2016 the French Ministry of Health announced universal access to DAA [13]. Before this announcement, initiating DAA treatment was subject to a multidisciplinary consultation meeting (RCP) which evaluated its relevance on a case by case basis [14]. Clinical criteria were usually the basis for selecting patients for DAA treatment. We do not know to what extent other patient characteristics, especially those that may limit patients’ acceptability of, engagement in, or adherence to treatment, influenced selection for DAA initiation [10,15]. Injecting drug use is a well described factor which can delay HCV treatment initiation [16]. It has also been reported that HCV-infected patients with psychiatric disorders are less likely to benefit from HCV treatment. In France, Roux et al [17] also reported that people who inject drugs (PWID) (currently or in the past) were less likely to be treated for HCV in the PEG-IFN era. However, a previous study using data collected during the ANRS CO13 HEPAVIH cohort showed no relationship between a history of injecting drug use and access to PEG-IFN+ ribavirin treatment [18]. While the HCV infection epidemic in France, and more generally in Europe, has mainly been driven by injecting drug use [19], recent data suggest the epidemiology of HCV infection in HIV patients is changing [20]. Given that national HCV guidelines strongly recommend access to HCV treatment for HIV patients [14] and that free access to DAA has been possible since May 2016 in France, we aimed to compare the socio-behavioral characteristics of patients initiating HCV treatment in the ANRS CO13 HEPAVIH cohort between those receiving PEG-IFN-based treatment and those receiving DAA, our hypothesis being that patients’ characteristics may play an important role in caregiver-patient relationships, as well as in access to and retention in the HCV and HIV care systems.

Our objective was not simply to identify possible barriers to accessing DAA treatment, but also to highlight the change in the profile of HIV-HCV co-infected patients initiating HCV treatment.

## Methods

### Study design and population

ANRS CO13 HEPAVIH is a multicenter observational cohort initiated in France in 2005 among HIV-HCV co-infected patients to characterize the natural history of co-infection in terms of both morbidity and mortality and their determinants, and to better understand the interactions between the two viruses and treatments [21]. ANRS CO13 HEPAVIH recruited outpatients from 32 clinical centers in France who were aged 18 or over, had both chronic hepatitis C and HIV infection, had detectable plasma HCV RNA, were positive for anti-HCV antibodies, and agreed to participate in the cohort (a signed letter of informed consent being provided).

Patient recruitment for this cohort included three phases:

- The first phase between December 2005 and December 2008 recruited adult HIV-positive patients, who were also either chronically infected by HCV at enrollment or had a negative HCV RNA test by PCR six months after ending HCV treatment (PEG-IFN),
- The second phase, from September 2011 to March 2016, recruited both individuals who spontaneously cleared HCV before the enrolment, and HIV-HCV infected patients who initiated HCV treatment with triple therapy (PEG-IFN +ribavirin + either telaprevir or boceprevir),
- The third phase, from May 2014 to November 2015, was an additional recruitment phase, where patients who were already receiving, or were scheduled to receive DAA within six months, were recruited.

In France, PEG-IFN+ ribavirin first became available in 2000. Boceprevir and Telaprevir became available in 2010. All three regimens were replaced from 2014 onwards with DAA.

**Fig 1:**
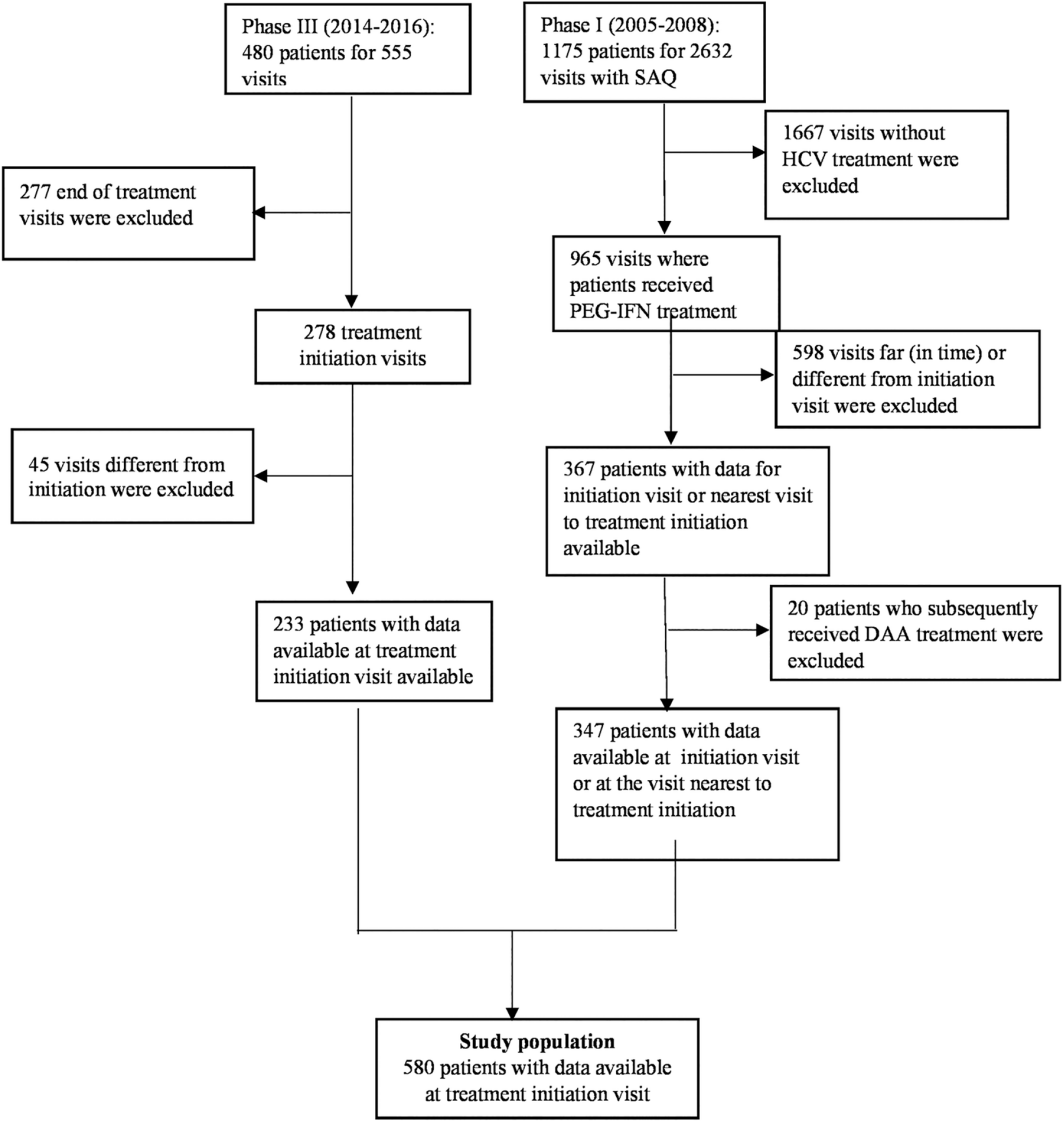
Flow chart of the patients included in the study.

In the present study, we included all cohort patients who had received or were still receiving HCV treatment, whether PEG-IFN-based in the first phase of the cohort or DAA-based in the third phase (Fig 1).

We preferred to exclude Phase 2 patients from this comparison for two reasons: first, a very small sample were eligible for this study, mainly patients in a severe state of illness and patients who were very unresponsive to Phase 1 treatment (ie PEG-IFN+Ribavirin) due to the high toxicity of this treatment. In addition, patients in the Phase 2 did not complete the annual self-administered questionnaires.

### Data collection

For patients included in the first recruitment phase, clinical visits were scheduled annually for non-cirrhotic patients and every six months for cirrhotic patients. Collection of socio-behavioral data using self-administered questionnaires (SAQ) was scheduled annually. For patients initiating HCV treatment, additional visits were scheduled, before, during and after the end of treatment. For patients included in the third recruitment phase receiving DAA, clinical and biological data were collected at treatment initiation, at the end of treatment, and six months after the end of treatment, using medical questionnaires. These data included weight, blood pressure, clinical stage of HIV infection, blood count, immuno-virological data, hepatic and renal assessment values, HCV RNA PCR measurements, and data on HCV and HIV treatments (drug, dosage, indication, initiation date and date of treatment end).

Socio-behavioral data were collected from SAQ at treatment initiation and at the end of treatment. The SAQ included items concerning patients’ socio-demographic characteristics and information on patients’ use of psychoactive drugs, tobacco, and alcohol (AUDIT-C).

### Variables

#### Alcohol consumption

Alcohol consumption was measured using the AUDIT-C questionnaire. This scale is composed of three questions each of which having four possible answers (i.e., a score ranging from 0 to 4; total scale score ranging from 0 to 12). Hazardous alcohol consumption was defined as having a AUDIT-C score ≥ 4 for men or ≥ 3 for women [22]. Binge drinking was defined as consuming six or more alcoholic drinks on any one occasion during the previous month.

#### Drug use

Patients were asked about their consumption of the following psychoactive substances during the previous four weeks: cannabis, cocaine, crack, heroin, ecstasy, amphetamines, LSD. With regard to cannabis consumption, patients were classified into three categories: “No use”, “non-regular use” and “regular or daily use.”

#### Tobacco consumption

Tobacco smoking (smoking status, history of smoking, number of cigarettes per day) was assessed during face-to-face interviews with physicians for patients in the first and second recruitment phases and using self-administered questionnaires for patients from the third phase.

#### Unstable housing conditions

Patients who were neither tenant nor owner of their housing were considered to have unstable housing.

For the evaluation of liver fibrosis, we used the FIB4 index obtained by the following formula [23]

$$Fib4\;index=\frac{Age\;(year)*AST\;(U/L)}{Platelets\;count\;{(10}^{9}/L)*\sqrt{ALT\;(U/L)}}$$

Severe fibrosis or cirrhosis was defined as a FIB-4 index score >3.25. A FIB-4 score between 1.45 and 3.25 corresponded to moderate fibrosis and <1.45 to no clinically significant fibrosis [24]. A FIB-4 score above 1.45 corresponded to significant fibrosis.

### Statistical analyses

Statistical analyses were performed using SAS software, version 9.4 (SAS Institute, Cary, North Carolina). The main characteristics of patients at HCV treatment initiation were compared between patients initiating PEG-IFN-based treatment (Phase 1) and those initiating DAA (Phase 3) (chi-square test for categorical variables, Student’s t test for continuous variables). We used data from the SAQ completed at the visit closest to the date of treatment initiation for patients receiving PEG-IFN treatment (Fig 1). For patients receiving DAA, we used data collected during the visit scheduled at the beginning of treatment.

A multivariable analysis was then performed with logistic regression models, using a binary variable indicating the type of treatment received (PEG-IFN vs DAA) as the dependent variable. All significant variables with a p-value of <0.20 in the univariate analyses were introduced into the multivariable regression model to estimate the adjusted effect and derive the adjusted odds ratio (aOR) for each of the dependent variables. A 95% confidence level was also estimated for each aOR.

In order to verify the robustness of the results and exclude possible recruitment phase bias, i.e. the possibility that patients recruited in the third phase had a different pattern of socio-behavioral characteristics from the patients in the first phase, a sensitivity analysis was conducted which included only patients followed up in clinical centers involved in all three phases of the cohort.

### Ethics statement

This study was approved by the "Comité de Protection des Personnes (CPP)" (Ref N° 2234 on April 19th, 2005) and the “Commission Nationale de l’Information et des Libertés de France (CNIL)” on September 6^th^, 2005. Participant provided signed, informed consent.

## Results

### Participants’ characteristics

A total of 580 HIV-HCV co-infected patients treated for chronic hepatitis C during the follow-up were included in the study (Fig 1). Mean age was estimated at 47.8 years±7.6 (range 23 to 71 years). Most were male (75.9%; 439/578) and just under a fifth (17.4%; 99/569) had unstable housing (Table 1).

**Table 1:** Comparison of patients’ socio-behavioral profiles at HCV treatment initiation in the ANRS CO13 HEPAVIH cohort (N = 580).

Patients’ characteristics	N (%)	Univariate analysis			Multivariable analysis
		PEG-IFN treatment N (%)	DAA treatment N (%)	p-value	aOR [95% CI]
**HCV treatment**	**580**	**-**	**-**	**-**	
*Peg-IFN*	347 (59.8)				
*DAA*	233 (40.2)				
**Age**	**572**	**347**	**225**	**0.0001**	**1.17 [1.13; 1.22]**
*Mean (SD)*	47.8 (7.6)	45.1 (6.2)	52.00 (7.8)		
*Median* (IQR)	48 (43–52)	45 (42–49)	52 (49–56)		
**Gender**	**578**	**347**	**231**	0.270	
*Male*	439 (75.9)	258 (74.4)	181 (78.4)		
*Fem*ale	139 (24.1)	89 (25.6)	50 (21.6)		
**Living in a couple**	**573**	**345**	**228**	0.835	
*Yes*	277 (48.3)	168 (48.7)	109 (47.8)		
*No*	296 (51.7)	177 (51.3)	119 (52.2)		
**Unstable housing**^**1**^	**569**	**345**	**224**	**0.0016**	
*Yes*	99 (17.4)	74 (21.4)	25 (11.2)		**0.46 [0.24; 0.88]**
*No*	470 (82.6)	271 (78.6)	199 (88.8)		**1**
**Plasma HIV-RNA <50 copies/ml**	**442**	**345**	**97**	**0.0003**	
*Yes*	372 (84.2)	279 (80.9)	93 (95.9)		
*No*	70 (15.8)	66 (19.1)	4 (4.1)		
**CD4 count–cells/mm**^**3**^	**442**	**342**	**100**	**0.0007**	
*Mean (SD)*	511 (278)	474 (251)	641 (325)		
*Median* (IQR)	462 (303–656)	430 (287–599)	628 (411–838)		
**HCV** viral load, log_10_ UI/ml	**364**	**265**	**99**	**<0.001**	
*Mean (SD)*	13.6 (2.1)	13.6 (2.3)	13.7 (1.5)		
*Median* (IQR)	13.9 (12.8–15.0)	14 (12.7–15.2)	13.8 (13–14.7)		
**Tobacco smoking**	**559**	**328**	**231**	0.0896	
*Yes*	364 (65.1)	223 (68.0)	141 (61.0)		
*N*o	195 (34.9)	105 (32.0)	88 (39.0)		
** Hazardous alcohol consumption**^**2**^	**557**	**347**	**210**	0.3866	
*Yes*	155 (27.8)	101 (29.1)	54 (25.7)		
*No*	402 (72.2)	246 (70.9)	156 (74.3)		
**Binge drinking**^**3**^	**555**	**344**	**211**	0.9362	
*Yes*	151 (27.2)	94 (27.3)	57 (27.0)		
*No*	404 (72.8)	250 (72.7)	154 (73.0)		
**Drug use**^**4**^	**580**	**347**	**233**	**0.0003**	
*Yes*	224 (38.6)	155 (44.7)	69 (29.6)		
*No*	356 (61.4)	192 (55.3)	164 (70.4)		
**Cannabis use**	**516**	**305**	**211**	**0.0002**	
*No use*	314 (60.9)	163 (53.4)	151 (71.6)		**1**
*Non- regular use*	95 (18.4)	68 (22.3)	27 (12.8)		**0.41 [0.22; 0.77]**
*Regular or daily use*	107 (20.7)	74 (24.3)	33 (15.6)		**0.50 [0.28; 0.91]**
**Cocaine use**	**580**	**347**	**233**	0.7647	
*Yes*	37 (6.4)	23 (6.6)	14 (6.0)		
*No*	543 (93.6)	324 (93.4)	219 (94.0)		
**Crack use**	**580**	**347**	**233**	0.6875	
*Yes*	4 (0.7)	2 (0.6)	2 (0.9)		
*No*	576 (99.3)	345 (99.4)	231 (99.1)		
**Heroin/subutex use**	**580**	**347**	**233**	0.1554	
*Yes*	17 (2.9)	13 (3.8)	4 (1.7)		
*No*	563 (97.1)	334 (96.3)	229 (98.3)		
**Ecstasy use**	**596**	**347**	**233**	0.1091	
*Yes*	19 (3.3)	8 (2.3)	11 (4.7)		
*No*	561 (96.7)	339 (97.7)	222 (95.3)		
**Amphetamines use**	**580**	**347**	**233**	**0.0302**	
*Yes*	6 (1.0)	1 (0.3)	5 (2.2)		
*No*	574 (99.0)	346 (99.7)	228 (97.8)		
**LSD use**	**580**	**347**	**233**	0.5345	
*Yes*	4 (0.7)	3 (0.9)	1 (0.4)		
*No*	576 (99.3)	344 (99.1)	232 (99,6)		
**Significant liver fibrosis**^**5**^	**502**	**343**	**159**	0.0293	
*Yes*	329 (65.5)	214 (62.4)	115 (72.3)		
*No*	173 (34.5)	129 (37.6)	44 (27.7)		
**Severe liver fibrosis or cirrhosis**^**5**^	**502**	**343**	**159**	0.049	
*Yes*	112 (22.3)	68 (19.8)	44 (27.7)		
*No*	390 (77.7)	275 (80.2)	115 (72.3)		
**Drug injection history**	**559**	**331**	**228**	<0.0001	
*Yes*	317 (56.7)	228 (68.9)	89 (39.0)		**0.19 [0.12; 0.31]**
*No*	242 (43.3)	103 (31.1)	139 (61.0)		**1**

^1^ Unstable housing was defined as not being owner or tenant of one’s housing.

^2^ Hazardous alcohol consumption was defined as having an AUDIT-C score ≥ 4 for men or ≥ 3 for women

^3^ Binge drinking was defined as consuming six or more alcoholic drinks on any one occasion during the previous month.

^4^ Drug use was defined as the use of at least one of the following psychoactive substances during the previous four weeks: cannabis, cocaine, crack, heroin, ecstasy, amphetamines, LSD.

^5^ Significant liver fibrosis was defined as a FIB-4 score >1.45, and severe fibrosis or cirrhosis as a FIB-4 score >3.25.

Of the total patient sample, 233 (40.2%) were on DAA. These individuals were significantly older than those who received PEG-IFN treatment (p = 0.0001) (Table 1). A significantly higher proportion of patients who received PEG-IFN treatment (21.4%) had unstable housing conditions (vs those receiving DAA (11.2%)) (p = 0.0016). (Table 1).

### Addictive behaviors

Just under two-thirds (65.1%; 364/559) of the study population were active tobacco smokers. No significant difference was observed between the two groups (Table 1).

Of the total study population, 27.8% (155/557) reported hazardous alcohol consumption and 27.2% (151/555) binge drinking. No significant difference was detected between the two groups of patients regarding hazardous alcohol consumption (29.1% among PEG-IFN-treated patients vs 25.7% among DAA-treated patients, p = 0.3866) and binge drinking (27.3% vs 27.0%, p = 0.9362) (Table 1).

In terms of drug use, two-fifths of the total population (38.6%; 224/580) were drug users. One-fifth were regular or daily cannabis users: 20.7% (107/516) (Table 1). Almost three-fifths (56.7%; 317/559) had a history of drug injection. Although, the proportion of patients who reported drug use was significantly higher in the PEG-IFN group (44.7% vs 29.6%, p = 0.0003), the consumption of drugs other than cannabis did not differ between both treatment groups (p = 0.98). The proportion of patients who reported regular cannabis consumption was significantly lower in the DAA group (15.6% vs 24.3%, p = 0.0002). Moreover, the proportion of patients with a history of drug injection was significantly higher in the PEG-IFN group (68.9% vs 39.0%; p<0.0001) (Table 1). Finally, the proportion of patients with a history of drug injection was significantly higher among those who had unstable housing than among those who did not (71.7% vs 54.4%, p = 0.0016).

In terms of clinical characteristics, two-thirds of the study population (65.5%) had significant liver fibrosis. The proportion of patients with significant fibrosis was higher in the DAA group (72.3% vs 62.4%, p = 0.0293) (Table 1).

### Multivariable analysis

In the multivariable analysis, only age, unstable housing, cannabis use and a history of drug injection remained associated with the type of HCV treatment received.

In our study sample, for each 1-year increase in age, patients were 17% more likely (aOR = 1.17 95% CI [1.13; 1.22]) to receive DAA treatment. In contrast, patients with unstable housing were less likely to receive DAA treatment (0.46 [0.24; 0.88]) than PEG-IFN based regimens. With respect to cannabis consumption (reference group: non-users), patients who used cannabis regularly or daily were 50% less likely to receive DAA treatment than PEG-IFN based regimens (0.50 [0.28; 0.91]), while non-regular users were 59% less likely (0.41 [0.22; 0.77]) to receive DAA treatment. Similarly, patients with a history of injecting drug use were 81% less likely to receive DAA treatment (0.19 [0.12; 0.31]) than to receive PEG-IFN based regimens (Table 1).

### Sensitivity analysis

In order to eliminate any possible selection bias arising from the fact that some centers followed a greater number of HIV-HCV co-infected patients who used drugs, we performed a sensitivity analysis which excluded clinical centers not involved in all 3 phases of the cohort, Accordingly, only 27 clinical centers were included in the sensitivity analysis which led to 26 patients (4.4%) being excluded. The results of the sensitivity analysis confirmed those of the main multivariable analysis (data not shown).

## Discussion

Our analysis of data from the ANRS CO13 HEPAVIH cohort is the first to compare characteristics of patients treated with first-generation HCV regimens with that of patients treated with DAA. Interestingly, patients with unstable housing and a history of drug injection (factors which both characterize vulnerable groups) and patients with regular cannabis use were all less likely to receive DAA treatment than PEG-IFN-based regimens. Moreover, the sensitivity analysis, which removed the possible recruitment effect, confirmed these results.

Given that there is no medical argument to delay providing a patient with DAA, as these treatments extremely effective and safe, universal access to DAA should help to better control the HCV epidemic [25]. In 2016, French policy makers stated universal access to HCV therapy, given that all patients with chronic hepatitis C at any stage of liver fibrosis, and from all vulnerable groups (i.e., HIV-HCV co-infected patients, people who use drugs, etc.), have the right to access HCV treatment and care according to the recommendations by the French High Authority in Health (HAS) (cf: http://www.has-sante.fr/portail/upload/docs/application/pdf/2016-05/aad_avis_25052016_ct_25052016.pdf).

Our primary focus was to understand whether there were any differences between HIV-HCV co-infected patients treated with DAA and those treated with PEG-IFN based regimens in terms of socio-behavioral characteristics.

The association we found between less access to DAA and unstable housing is consistent with prior studies [15] which reported that unstable housing is a barrier to initiation of chronic hepatitis C treatment, as it could compromise adherence to treatment. People with unstable housing are also more likely to have poorer financial and psychosocial statuses. Therefore, they are not only at a high risk of HCV infection [26], but are also exposed to discrimination, social isolation and sometimes rejection. Given this, the fear of rejection might affect patient-provider relationships, and this may not regularly seek medical care and often miss follow-up visits in HIV medical centers. Patients with unstable housing are known to be less likely to have a long-term commitment to continued medical care for HIV and other comorbidities, including HCV infection [27]. There is also the risk that some health workers mistrust of patients may contribute to delayed access to care, including HCV treatment with DAA. In addition to factors related to the price of DAA, to caregivers and to the healthcare system itself [28,29], in our study, it is possible that the majority of HIV-HCV co-infected patients with unstable housing conditions were treated and cured in the era of PEG-IFN. That could be the result of policy efforts to treating vulnerable populations.

PWID constitute a key group where the prevalence of chronic hepatitis C is high, due to shared injection equipment [30]. In French and international (cf http://www.eacsociety.org/files/guidelines_8_0-english_web.pdf) guidelines, HCV treatment with DAA is strongly recommended for HIV-HCV co-infected patients and drug users, irrespective of disease stage, as it constitutes an important tool for prevention of HCV transmission. Previous studies on HCV mono-infected and HIV-HCV co-infected PWID showed high rates of sustained virological response (SVR) to HCV treatment in these populations, and also showed that PWID did not exhibit lower rates of adherence to treatment than other patients [31–33]. Another issue with PWID is the risk of reinfection after SVR to HCV treatment. Despite the relatively low rate of reinfection among co-infected patients, estimated at 1%-5%, in one study [34], this rate can still have an impact at the population level and compromise the global eradication of HCV [35]. Although one study highlighted that HIV-HCV co-infected PWID in France are not less likely to be treated with PEG-IFN based regimens [18], another French study [17] found the opposite. Our results showed that this population is less represented among DAA-treated patients, which could suggest they have less access to this new treatment [36]. In a Canadian HIV-HCV co-infected cohort, Saeed et al. [37], found great disparities in DAA initiation, and highlighted that marginalized populations, including PWID, were less likely to initiate HCV treatment in the DAA era.

Some physicians may tend to delay HCV treatment in active users of psychoactive substances, including cannabis, because of concerns about poor adherence to treatment and the high risk of re-infection after treatment [38,39]. However, research has shown that patients using cannabis often do so to relieve a range of troublesome side effects of PEG-IFN-treatment [40]. In addition, regular cannabis use is frequent in former injecting drug users and more particularly in former opioid-dependent individuals, as it relieves opioid withdrawal symptoms and HIV-HCV-related symptoms [41,42]. In the present study, we also found that the proportion of cannabis users was lower in individuals receiving DAA, suggesting either that cannabis users had already been treated with PEG-IFN based treatment or that cannabis users have reduced access to DAA. Cannabis use is an additional proxy of history of drug use or unstable lifestyles. Most drug users in our study were treated with PEG-IFN or with DAA. Yet, to be effective, the management of these patients has to go beyond simply providing medical treatment. Substance abuse requires integrated, multidisciplinary care. Promoting stable housing through a "housing first" approach also seems to be crucial [43,44]. The results of this study also showed that older patients were more likely to receive DAA treatment than PEG-IFN–based regimens. This population included individuals who did not respond to PEG-IFN, and others whose advanced stage of liver disease (advanced fibrosis or cirrhosis, hepatocellular carcinoma) was a criterion to schedule treatment. This was highlighted by the difference in liver fibrosis found when comparing the two groups in univariate analysis. According to the natural history of chronic hepatitis C in patients infected with HIV, the occurrence of severe complications, including liver fibrosis or cirrhosis, is strongly associated with patient age [42] and this could influence HCV treatment SVR rates [45,46].

The results from our cohort study need to be further explored in a qualitative manner, from the perspective of both patients and caregivers [19], in order to better identify their beliefs and perceptions and to acquire a greater understanding of the reasons for delayed DAA initiation in some groups. The sensitivity analysis confirmed results from the main multivariable analysis. Current guidelines for access to HCV treatment in France stipulate that HIV-HCV co-infected patients and all vulnerable populations, including PWID, should be treated. However, our results suggest that this is not yet a reality. Indeed, since DAA are more effective and less toxic than PEG-IFN-based treatments, they should be accessible to more PWID, especially those who are most socially vulnerable, as this population is the reservoir of the HCV epidemic, facilitating HCV transmission in their network through risky practices [34].

Overall, the high prices which pharmaceutical firms ask for DAA may influence health policy decisions, and force clinicians to delay treatment initiation until liver fibrosis is already quite advanced. That could explain the fact that vulnerable and marginalized populations were less represented in patients receiving DAA. Such decisions are unacceptable now that chronic hepatitis C is a curable disease and that DAA have been proven to be cost-effective [47]. Before the advent of DAA, access to treatment for chronic hepatitis C was very selective in France, with priority being given to patients at an advanced stage of the disease and those with complications including liver fibrosis and hepatocellular carcinoma. It was the consensus that everyone has the right to benefit from HCV medical care [48] which led to the French government adopting a policy of universal HCV care in May 2016.

Overall, the results of this study highlighted a change over time in the profile of HIV-HCV co-infected patients treated for HCV infection in France. This evolution reflects improvements in the treatment of hepatitis C in France, as patients from the most vulnerable populations can now access treatment and therefore be cured.

Our study has limitations. First, we compared access to HCV treatment between patients treated at two different calendar periods, and consequently there may have been changes in the epidemiological characteristics of patients recruited. However, to tackle this potential bias, we performed a sensitivity analysis which confirmed the results found. Second, behaviors were based on self-reports which can be affected by social desirability bias. Nevertheless, even if some under-reporting were present, it is unlikely that this would vary over time and affect the comparison between access to treatment in the PEG-IFN and DAA period. Finally, given that is an observational cohort, it may be not appropriate to extrapolate these findings to all HIV-HCV co-infected patients in France.

## Conclusion

In this study, people with unstable housing conditions, those with a history of drug injection and cannabis users were all less represented among patients who initiated DAA treatment than among those who initiated PEG-IFN-based regimens. It is possible that a majority of these patients had previously been treated for HCV in the PEG-IFN era. This reflects a change in the epidemiological profile of HIV-HCV co-infected patients receiving HCV treatment in France. As HCV treatment is prevention, improving access to DAA, in particular to individuals with high-risk behaviors, remains a major clinical and public health strategy.
